# Terminal efficiency of Peruvian university students in the second specialty programs of a dental school over seven years

**DOI:** 10.12688/f1000research.157705.1

**Published:** 2024-10-31

**Authors:** Karen Llajaida Cabanillas-Yllesca, Karla Lucero Avalos-Baltodano, Roberto A. León-Manco, María Claudia Garcés-Elías, Cesar D. Rojas-Senador

**Affiliations:** 1 Facultad de Estomatología, Universidad Peruana Cayetano Heredia, Lima, Peru

**Keywords:** Education, Efficiency, Students, Dentistry, Universities

## Abstract

**Background:**

Concerns regarding academic efficiency at the postgraduate level in university programs require attention from those responsible for making university administrative decisions. This study aimed to determine the terminal efficiency of Peruvian university students enrolled in second specialty programs in a dental school over seven years.

**Methods:**

This cross-sectional study considered all records of documents submitted for obtaining a title of second professional specialty in the programs offered by a dental school, published in its institutional repository between 2017 and 2023. The study variable was terminal efficiency (TE), considered both as a quantitative and qualitative variable. Additionally, some covariates were included, such as the year of formal document submission, the mode of document preparation, the second professional specialty in dentistry, and sex. Descriptive analysis was conducted to obtain absolute and relative frequencies. Bivariate analysis was performed using Chi-square, Mann-Whitney U, and Kruskal-Wallis tests.

**Results:**

From the evaluation of TE between 2017 and 2023, it was observed that 72.09% (n=186) of students with a document submitted for obtaining a title of second professional specialty and published in the repository had a TE categorized of “Up to 1 year”. Likewise, the mean TE was 0.95 years (SD=1.25), with a statistically significant difference (p<0.001) between medians when evaluated based on the year of formal document submission and the dental specialty program.

**Conclusion:**

The mean terminal efficiency of Peruvian university students in second specialty programs in a dental school over seven years was 0.95 years.

## Introduction

Higher education, at its various levels, plays an important role in the training and specialization of professionals that countries demand; likewise, it constitutes a fundamental pillar for the progress of nations, due to the scientific-technical and cultural potential that frames these educational processes. Therefore, it is undeniable that higher education represents the main mechanism through which human capital acquires a set of both individual and collective competencies that contribute to the efficient performance of professionals in their respective fields of action. ^1^ In this context, through teaching and research, universities provide the education and innovation that professionals and their countries require to achieve consistent development. ^2^

The execution of activities within universities is closely linked to the constant pursuit of improving educational quality. In this sense, it becomes evident that these institutions need to plan strategies that allow them to enhance their performance, based on the establishment of educational and academic management indicators. ^3^ Considering that efficiency is a concept associated with an optimal level of functionality and the ability to achieve goals, the educational quality of a university can be assessed by determining its academic efficiency both qualitatively and quantitatively. ^4^ Thus, the indicator commonly used for this evaluation is called terminal efficiency (TE), classically described as the proportion of students who enter the educational system and complete the subjects it encompasses within the expected time, ^5, 6^ and previously determined by the structure of the academic program pursued by the university student.

University higher education in health sciences has increased in demand in recent years. Several studies have highlighted the need for universities offering these academic programs to understand the efficiency and effectiveness of the educational activities they develop, considering that the outcomes of university management can be reflected in the mean number of years a student invests in completing an academic program and obtaining a professional title. Thus, academic management indicators, such as TE, allow the quality of universities and their various programs to be assessed, and associated follow-up studies enable the evaluation of training intending to design proposals focused on improving these indicators. ^7^

Concerns about academic efficiency at the university level should not be exclusive to undergraduate academic programs; the conditions in which postgraduate programs are developed also need to be addressed by those responsible for making university administrative decisions. This stems from the recognition of the difficulty in achieving an optimal level of quality in an academic program, particularly in the context of professional specialization. TE represents a complex issue, and at the same time, one that is under-researched in postgraduate education. However, it is necessary to understand it in light of the latent increase in the availability of professional specialization programs, whose quality may be affected by various factors, such as the selection of professionals capable of meeting current societal needs and demands, among others that require a strategic approach from universities. ^8^

It has been shown that university students who complete an academic program often do not obtain a professional title due to economic, social, cultural factors, or even as a result of inadequate management of research, which may be incomprehensible to the student or related to the use of formats that cause delays in the process of preparing the various documents required to obtain a professional title. ^9^ Thus, ensuring that university students obtain a professional title that validates their specialization studies and prepares them to achieve the desired performance in a highly competitive job market is of vital importance. Therefore, this professional title should be obtained in the shortest time possible, and it is the responsibility of the university to contribute to this, as its ability to do so represents a key measure of its academic efficiency. In this regard, the present study aimed to determine the terminal efficiency of Peruvian university students in second specialty programs of a dental school over seven years.

## Methods

### Study design

For this study, a cross-sectional design was proposed, with the population consisting of all document records submitted for obtaining a title of second professional specialty in each of the programs offered by the Postgraduate and Specialization Unit of the Facultad de Estomatología de la Universidad Peruana Cayetano Heredia (UPCH) in Lima, Peru, published in the institutional repository between 2017 and 2023. The decision was made to work with the entire population; therefore, no sample size was determined.

The study encompassed records of all students enrolled in second professional specialty programs at UPCH from 2017 to 2023, subject to specific selection criteria. Inclusion required complete documentation of program admission date, academic status, and graduation date for those who completed the program. Conversely, records with incomplete or inconsistent information in the above fields were excluded. Furthermore, the study omitted records of exchange students and those who transferred from other universities during the specified period.

### Variables

This research considered terminal efficiency (TE) as both a quantitative and qualitative variable. Additionally, some covariates were included, such as the year of formal document submission, the mode of document preparation, the second professional specialty in dentistry, and sex.

TE, as a quantitative variable or mean TE, was calculated from the difference in years between the formal submission of the document to obtain the title of second professional specialty to an expert jury, verified with the publication in the institutional repository of UPCH, and the student’s graduation year. As a qualitative variable or categorized TE, two categories were considered: “Up to 1 year” when TE was less than or equal to 1, and “2 years or more” when TE was greater than or equal to 2.

The mode of document preparation, as a qualitative variable, considered two categories: “Individual” when the document to obtain the title of second professional specialty in dentistry was prepared by a single student, and “Collective” when the document was prepared by two students or more.

The second professional specialty in dentistry, as a qualitative variable, considered twelve categories: “Dental Auditing”, “Oral and Maxillofacial Surgery”, “Endodontics”, “Special Patients Stomatology”, “Integral Oral Implantology”, “Restorative and Esthetic Dentistry”, “Pediatric Dentistry”, “Orthodontics and Maxillary Orthopedics”, “Periodontics and Implantology”, “Oral and Maxillofacial Radiology”, “Oral Rehabilitation”, and “Dental Public Health” which are part of the Peruvian list of recognized dental specialties in Article 26th of the Regulation of the Dentist’s Labor Law, Law No. 27878, amended in 2020 by Supreme Decree No. 023-2020-SA. ^10^

### Data collection

Based on the information published in the UPCH institutional repository during the period 2017–2023, data such as the sex of Peruvian university students in each of the specialty programs were obtained. Meanwhile, the year of formal document submission, the mode of document preparation, and the second professional specialty in dentistry were gathered from the corresponding documents submitted to obtain the title of second professional specialty. Subsequently, the UPCH institutional repository was requested to provide information regarding the graduation year of students with a document published in the repository for obtaining the title of second professional specialty, which was necessary for calculating the mean TE, from which the categorized TE was determined. Finally, all the collected data were organized to facilitate subsequent analysis.

### Data analysis

The descriptive analysis provided absolute and relative frequencies for the entire population because there were no missing data. For the bivariate analysis, associations were evaluated using the Chi-square statistical test; additionally, the Kolmogorov-Smirnov test was used to assess the normality of the data distribution, and the non-parametric Mann-Whitney U and Kruskal-Wallis tests, with the corresponding post hoc test, were employed to determine differences between groups. The study considered a 95% confidence level and a p-value <0.05 to determine statistical significance. STATA v. 18.0 software was used to perform the various analyses, and Microsoft Excel 365 was used to organize and present the results in tables.

Sensitivity analyses were conducted to ensure the robustness of the findings, given that non-parametric statistical methods were employed due to the non-normal distribution of the data. Since the entire population of eligible records was included, no sampling bias was expected. However, additional analyses were performed by recalculating key outcomes after excluding potential outliers to confirm the stability of the results. These analyses demonstrated that the main findings regarding TE remained consistent, further reinforcing the reliability of the conclusions despite the use of non-parametric methods.

In this study, several efforts were made to address potential sources of bias. First, to minimize selection bias, all available records of documents submitted for obtaining a second professional specialty title between 2017 and 2023 were included, ensuring comprehensive data collection. Additionally, the study controlled for temporal bias by including the year of formal document submission as a covariate in the analysis, accounting for any year-specific variations. Furthermore, bivariate analyses were performed using appropriate statistical tests (Chi-square, Mann-Whitney U, and Kruskal-Wallis), ensuring that differences between subgroups were adequately examined.

## Results

Between 2017 and 2023, 258 university students from the second specialty programs of a dental school at a Peruvian university had a document submitted to obtain their corresponding title of second professional specialty published in the institutional repository of UPCH. In 2020, 20.92% of students (n=54) had a published document, representing the year with the highest percentage, while 2017 had the lowest percentage, as only 9.69% (n=25) of students had a document published in the repository. A total of 60.47% (n=156) of students individually prepared their document to obtain their title of second professional specialty, while 39.53% (n=102) did so collectively. When considering the different second professional specialties in dentistry, it was found that the highest percentage of students, 20.54% (n=54), with a document submitted to obtain their title of second professional specialty and published in the repository belonged to the Oral and Maxillofacial Radiology program, while the lowest percentage of students, 0.78% (n=2), belonged to the Dental Auditing and Integral Oral Implantology programs. Additionally, 60.85% (n=157) of the students with a document in the repository were male, while 39.25% (n=101) were female ( Tables 1 and 2).

**Table 1.** Categorized terminal efficiency of Peruvian university students in the second specialty programs of a dental school over seven years.

Variables	n	%	Terminal efficiency (Categorized)				
			Up to 1 year		2 years and older		p
			n	%	n	%	
Total	258	100.00	186	72.09	72	27.91	
Year of formal document submission							
2017	25	9.69	21	84.00	4	16.00	0.057 *
2018	33	12.79	30	90.91	3	9.09	
2019	42	16.28	25	59.52	17	40.48	
2020	54	20.92	39	72.22	15	27.78	
2021	39	15.12	28	71.79	11	28.21	
2022	30	11.63	19	63.33	11	36.67	
2023	35	13.57	24	68.57	11	31.43	
Mode of document preparation							
Individual	156	60.47	111	71.15	45	28.85	0.677 *
Collective	102	39.53	75	73.53	27	26.47	
Second professional specialty in dentistry							
Dental Auditing	2	0.78	2	100.00	0	0.00	0.544 **
Oral and Maxillofacial Surgery	8	3.10	3	37.50	5	62.50	
Endodontics	28	10.85	22	78.57	6	21.43	
Special Patients Stomatology	7	2.71	5	71.43	2	28.57	
Integral Oral Implantology	2	0.78	1	50.00	1	50.00	
Restorative and Esthetic Dentistry	23	8.91	18	78.26	5	21.74	
Pediatric Dentistry	31	12.02	24	77.42	7	22.58	
Orthodontics and Maxillary Orthopedics	21	8.14	18	85.71	3	14.29	
Periodontics and Implantology	18	6.98	6	33.33	12	66.67	
Oral and Maxillofacial Radiology	53	20.54	48	90.57	5	9.43	
Oral Rehabilitation	36	13.95	21	58.33	15	41.67	
Dental Public Health	29	11.24	18	62.07	11	37.93	
Sex							
Male	157	60.85	112	71.34	45	28.66	0.736 *
Female	101	39.15	74	73.27	27	26.73	

n: Absolute frequency; %: Relative frequency; p: Statistical significance.

* Chi-square test.

** Chi-square test corrected by Yates.

**Table 2.** Mean terminal efficiency of Peruvian university students in the second specialty programs of a dental school over seven years.

Variables	n	%	Terminal efficiency (in years)				
			X	SD	M	IQR	p
Total	258	100.00	0.95	1.25	1.00	2	
Year of formal document submission							
2017	25	9.69	0.48	1.08	0.00abcd	1	<0.001 *
2018	33	12.79	0.33	0.78	0.00efghi	1	
2019	42	16.28	1.14	0.93	1.00aej	2	
2020	54	20.92	0.74	1.01	0.00fjk	2	
2021	39	15.12	1.08	1.24	1.00bg	2	
2022	30	11.63	1.50	1.68	1.00chk	2	
2023	35	13.57	1.37	1.61	1.00di	3	
Mode of document preparation							
Individual	156	60.47	0.94	1.37	0.00	2	0.171 **
Collective	102	39.53	0.97	1.05	1.00	2	
Second professional specialty in dentistry							
Dental Auditing	2	0.78	1.00	0.00	1.00a	0	<0.001 *
Oral and Maxillofacial Surgery	8	3.10	2.00	1.60	2.00bcde	4	
Endodontics	28	10.85	0.75	1.00	1.00bfgh	1	
Special Patients Stomatology	7	2.71	1.43	1.51	1.00i	3	
Integral Oral Implantology	2	0.78	1.50	0.71	1.50j	-	
Restorative and Esthetic Dentistry	23	8.91	0.57	1.31	0.00cklmn	1	
Pediatric Dentistry	31	12.02	1.03	1.20	1.00kop	1	
Orthodontics and Maxillary Orthopedics	21	8.14	0.62	1.20	0.00dqr	1	
Periodontics and Implantology	18	6.98	2.00	1.37	2.00floqst	2	
Oral and Maxillofacial Radiology	53	20.54	0.30	0.97	0.00aegijpsuv	0	
Oral Rehabilitation	36	13.95	1.31	0.82	1.00hmrtu	1	
Dental Public Health	29	11.24	1.28	1.49	1.00nv	2	
Sex							
Male	157	60.85	0.98	1.25	1.00	2	0.512 **
Female	101	39.15	0.91	1.26	0.00	2	

n: Absolute frequency; %: Relative frequency; X: Mean; SD: Standard deviation; M: Median; IQR: Interquartile range; p: Statistical significance.

* Kruskal-Wallis test; post hoc Mann Whitney U test, equal letters show a statistically significant difference (p<0.05).

** Mann Whitney U test.

From the evaluation of TE, it was observed that 72.09% (n=186) of students with a document submitted to obtain their title of second professional specialty published in the repository had a categorized TE of “Up to 1 year”. In 2020, the highest number of students (n=39) with a categorized TE of “Up to 1 year” was recorded, representing 72.22% of students who had a document in the repository that year. On the other hand, in 2022, the lowest number of students (n=19) with a categorized TE of “Up to 1 year” was observed, corresponding to 63.33% of students who had a document published in the repository that year. Among the students who individually prepared their documents, 71.15% (n=111) achieved a categorized TE of “Up to 1 year”; meanwhile, in the group of students who collectively prepared their documents, 73.53% (n=75) also achieved a categorized TE of “Up to 1 year”. Regarding the different second professional specialties in dentistry, the highest number of students (n=48) with a categorized TE of “Up to 1 year” was recorded in the Oral and Maxillofacial Radiology program, representing 90.57% of students who had a document published in the repository in that academic program. Conversely, the lowest number of students (n=1) with a categorized TE of “Up to 1 year” was recorded in the Integral Oral Implantology program, corresponding to 50.00% of students who had a document published in the repository in that academic program. Among male students with a document published in the repository, 71.34% (n=112) achieved a categorized TE of “Up to 1 year”, while 73.27% (n=74) of female students also achieved a categorized TE of “Up to 1 year” ( Table 1).

Similarly, between 2017 and 2023, the mean TE of students with a document submitted to obtain their title of second professional specialty published in the institutional repository was 0.95 years (SD=1.25). The lowest mean TE was recorded in 2018, with 0.33 years (SD=0.78), while 2022 had the highest mean TE with 1.50 years (SD=1.68). Among students who individually prepared a document, the mean TE was 0.94 years (SD=1.37); meanwhile, students who prepared a document collectively achieved a mean TE of 0.97 years (SD=1.05). The Oral and Maxillofacial Radiology program showed the lowest mean TE, with 0.30 years (SD=0.97), while the Oral and Maxillofacial Surgery and Periodontics and Implantology programs were the second professional specialties in dentistry with the highest mean TE, both at 2.00 years (SD=1.60 and SD=1.37, respectively). Male students with a document published in the repository achieved a mean TE of 0.98 years (SD=1.25), while female students achieved a mean TE of 0.91 years (SD=1.26). A statistically significant difference (p<0.001) was found between medians when evaluated by the year of formal document submission and the second professional specialties in dentistry ( Table 2).

## Discussion

TE is an indicator that evaluates university academic efficiency at the undergraduate and postgraduate levels, in terms of obtaining academic degrees and professional titles. It also allows the identification of the direct impact that the generation of knowledge through research activities, such as the preparation of academic papers or theses, has on the scientific and technological development of a country. ^11^ The positioning of TE as one of the main indicators to be considered by universities in a continuous evaluation process of educational quality highlights the existence of a relationship between institutional performance and the fulfillment of the objectives set by each university. ^12^ Thus, understood as an important part of the university educational-productive process, TE allows the determination of a university’s ability to professionally train some individuals within a specific time frame, considering that by certifying or accrediting them as professionals within that period, the university has fulfilled its objective. ^5^ Likewise, this process makes it possible to distinguish between university students who graduate and those who obtain a professional title, and this distinction, in turn, highlights that this indicator represents an important criterion for allocating budgets that will subsequently enable universities to have the necessary resources to promote high-quality professional training, empowering future graduates by helping them obtain their corresponding professional title. ^5, 13^

Despite its importance and the evident need for standardization in its calculation over time, TE has generated a series of conceptualizations. Notably, the definitions described in the studies by Pérez J and Cuéllar et al. define TE as the proportion of students who enter the educational system and complete the degrees it encompasses within the expected timeframe. ^1, 14^ López et al. define TE as a percentage ratio between the total number of graduates from a study program and the total number of students who entered the program “x” years earlier, with “x” being the number of years proposed for the completion of the study program. ^15^ Girano-Arévalo et al. define TE as the time elapsed between the completion or graduation from a university study program and the attainment of the corresponding professional degree through one of the various modalities considered by the university for this process. ^16^

This study adopted the TE indicator evaluation methodology proposed by Girano-Arévalo et al., who, in a 2021 study, determined the TE of undergraduate students from a Peruvian dental school between 1975 and 2018. They found that graduates with a document submitted to obtain their professional title published in the institutional repository achieved a mean TE of 1.67 years, and 60.51% of them showed a categorized TE of “Up to 1 year”, ^16^ both of which are less favorable values for the TE indicator compared to those obtained in the present study. This difference between the two studies could primarily be associated with the level of study of the populations, undergraduate and postgraduate respectively, considering that the level of commitment to personal goals generally differs between these populations. Likewise, the periods considered for both studies were different, and the development of the teaching-learning process may have been influenced by various social, political, and economic contexts, among others. Thus, although there are no ideal reference values for the TE indicator, a period of “Up to 1 year” for categorized TE is proposed as an appropriate criterion, suggesting that a shorter time associated with the mean TE is considered favorable. ^16^ Based on this clarification, it can be asserted that the values obtained for the TE indicator of Peruvian university students in second specialty programs at a dental school over seven years demonstrate an adequate level of functionality, but there is still room for optimization with the adoption of a series of well-directed measures.

Internationally, universities have proposed various modalities for obtaining a professional degree, including: thesis, overall grade point average, national professional quality exams, writing reports, comprehensive exams, participation in seminars or degree courses, writing research projects, developing textbooks, among others. Although it is clear that multiple options exist for obtaining a professional degree, the values reported when evaluating TE generate concern. ^11^

In Peru, since 2014, Law No. 30220 – the University Law – states that to obtain a second professional specialty degree, a bachelor’s degree or another equivalent professional title is required, along with the completion of studies with a minimum duration of two academic semesters and a minimum of forty credits, as well as the approval of an academic paper or a thesis. ^17^ Thus, the attainment of a second professional specialty degree is based on the strict fulfillment of the academic requirements established by universities in their internal regulations. In compliance with the current legal framework, UPCH establishes the following modalities for obtaining a second professional specialty degree in each of its programs: the academic paper and the thesis. ^18^ It is worth noting that the University Law lacks definitions that provide a specific delineation of each modality, ^17^ which could make it difficult for students to choose between one or the other. In contrast, the internal documents of UPCH clearly delineate these modalities, detailing the inclusion of the research project within the scope of the academic paper modality. ^18^ However, despite the existence of these alternatives, all documents formally submitted to an expert jury at UPCH for obtaining a second professional specialty degree in dentistry during the study period corresponded to the thesis modality.

While it is true that university activities are primarily focused on students, the number of diagnostic studies aimed at identifying the potential causes of unfavorable values for the TE indicator is limited, even more so at the postgraduate level, and specifically in second professional specialty programs in health sciences, such as dentistry. An important factor associated with these TE values is the delay in preparing the documents required to obtain a second professional specialty degree, which could be attributed to sociodemographic characteristics, personality traits, or even institutional factors, such as the research management procedures within the university environment. ^19^ Another critical aspect to consider is the impact that the COVID-19 pandemic had on university education, including the modification of teaching methodologies and the administrative and management procedures of universities to ensure the continuity of activities in this context. This particular period highlighted the importance of information and communication technologies. ^20^

Although the absence of specific studies evaluating the TE indicator in second professional specialty programs in dentistry that would allow comparisons with the present study, and the assessment of only some of the previously mentioned aspects, represent the limitations of this study, it is essential to highlight that directing actions towards developing processes that are suited to the characteristics of university students in second specialty programs in dentistry, which contribute to achieving satisfactory results in the evaluations of different academic efficiency indicators, requires greater support. It is important to note that the responsibility of universities is not limited to providing knowledge to their students but also includes generating a social contribution. This contribution can be reflected in the scientific, technological, and innovative development of the various areas that contribute to the progress of a country. Therefore, the training of professionals specialized in the different areas of dentistry requires the development of research and processes that reinforce the various competencies associated with the field. ^20^

In conclusion, the mean terminal efficiency of Peruvian university students in second specialty programs at a dental school over seven years was 0.95 years, representing a favorable value for the studied indicator.

### Ethical considerations

The study protocol was approved by the UPCH Institutional Research Ethics Committee (ethics file CONSTANCIA-CIEI-428-39-23, approved on October 2, 2023).

### Consent to participate

Consent to participate was not required, due to the study used secondary data, which was anonymized, ensuring that such modification does not distort the scientific meaning of the information. An accredited committee, the Universidad Peruana Cayetano Heredia Institutional Research Ethics Committee approved the study protocol in the “Exempt” category, which exempts the study protocol from expedited review and the need for consent to participate because the study uses information that is public in the university’s institutional repository.

## Author contributions

Karen Llajaida Cabanillas-Yllesca

Roles: Conceptualization, Methodology

Karla Lucero Avalos-Baltodano

Roles: Investigation, Resources

Roberto Antonio León-Manco

Roles: Conceptualization, Methodology, Writing – Review & Editing

María Claudia Garcés-Elías

Roles: Conceptualization, Methodology, Writing – Review & Editing

Cesar David Rojas-Senador

Roles: Data Curation, Formal Analysis, Investigation, Writing – Review & Editing

## Data Availability

Zenodo: Terminal efficiency of Peruvian university students in the second specialty programs of a dental school over seven years - Dataset. https://doi.org/10.5281/zenodo.13901816. ^22^ The project contains the following underlying data:
• Dataset-Terminal efficiency.xls. (Anonymised and codified data, with legend). Data are available under the terms of the Creative Commons Attribution 4.0 International license (CC-BY 4.0). Zenodo: STROBE checklist for “Terminal efficiency of Peruvian university students in the second specialty programs of a dental school over seven years”. https://doi.org/10.5281/zenodo.13939895. ^21^ Data are available under the terms of the Creative Commons Attribution 4.0 International license (CC-BY 4.0).
