# Terminal efficiency of Peruvian university students in the second specialty programs of a dental school over seven years

**DOI:** 10.12688/f1000research.157705.2

**Published:** 2025-05-09

**Authors:** Karen Llajaida Cabanillas-Yllesca, Karla Lucero Avalos-Baltodano, Roberto A. León-Manco, María Claudia Garcés-Elías, Cesar D. Rojas-Senador

**Affiliations:** 1 Facultad de Estomatología, Universidad Peruana Cayetano Heredia, Lima, Peru

**Keywords:** Education, Efficiency, Students, Dentistry, Universities

## Abstract

**Background:**

Terminal efficiency (TE), the proportion of students completing academic programs within the expected timeframe, is a critical metric for assessing postgraduate health education quality, yet it remains underexplored in Latin American dental specialties. Efficient training is vital for producing competent professionals to meet regional healthcare demands. This study aimed to determine the TE of Peruvian university students in second specialty dental programs at a Peruvian university from 2017 to 2023.

**Methods:**

A longitudinal, cross-sectional analysis was conducted using 257 complete records from the university institutional repository. Inclusion criteria encompassed full documentation of admission, academic status, and graduation dates. TE was calculated as the time from document submission to graduation, categorized as ≤1 year or ≥2 years. Statistical analyses included Chi-square tests, non-parametric Mann-Whitney U and Kruskal-Wallis tests, Poisson regression, and hierarchical multiple linear regression on log-transformed TE.

**Results:**

Mean TE was 0.93 years (SD=1.22), with 72.37% of students (n=186) completing within ≤1 year. Significant variations were observed by submission year (p=0.001) and specialty (p<0.001), with Oral and Maxillofacial Radiology (0.30 years) outperforming Periodontics and Implantology (1.76 years). The submission year was the only significant predictor of mean TE (β=0.144, p<0.001).

**Conclusion:**

Dental programs of a Peruvian university demonstrate robust TE, though specialty-specific and temporal disparities highlight areas for improvement. Targeted interventions, such as streamlined thesis processes and specialty-specific support, could enhance efficiency, informing educational policy in Latin America.

## Introduction

Higher education in health sciences is essential for training specialized professionals who contribute to national development through scientific, technical, and cultural advancements. Universities serve as key institutions in this process, providing the education and innovation required to equip professionals with the competencies needed to excel in their fields and address societal demands. ^1^ In Peru, the demand for advanced education in health sciences, particularly dentistry, has risen sharply in recent years, reflecting the growing need for skilled practitioners in a competitive healthcare landscape. ^2^ This trend underscores the critical importance of efficient academic programs that enable students to complete their training and obtain professional titles promptly, thereby enhancing their ability to contribute to public health and national progress.

Efficiency in higher education is a multifaceted concept, often evaluated through indicators that assess the quality and timeliness of academic outcomes. Among these, terminal efficiency (TE) stands out as a vital metric, defined as the proportion of students who complete their academic programs and obtain their professional titles within the expected timeframe. ^3^ In postgraduate settings, where specialized training is both intensive and time-sensitive, TE serves as a key measure of program effectiveness, reflecting the ability of institutions to prepare graduates for professional practice. ^4^ In dental education, TE is particularly significant, as it indicates how well specialty programs produce competent professionals capable of meeting complex oral health challenges.

Despite its importance, research on TE in postgraduate dental programs remains limited, especially in Peru and the broader Latin American context. While studies have explored TE at the undergraduate level, postgraduate programs—such as second specialty programs in dentistry—have received scant attention. ^5^ These programs are critical for developing advanced expertise, yet they face unique challenges, including delays in degree completion due to research requirements, institutional processes, or external factors like the COVID-19 pandemic. ^6^ In Peru, where the University Law (Law No. 30220) mandates specific academic requirements for second specialty degrees, including a minimum of two semesters with a minimum content of forty academic credits and a thesis or academic paper, understanding TE is essential for optimizing educational quality and addressing regional gaps in the literature. ^7^

This study aims to determine the terminal efficiency of Peruvian university students enrolled in second specialty programs at the dental school of a Peruvian university over a seven-year period (2017–2023). The seven-year timeframe was selected to capture trends and assess the impact of external disruptions, such as the COVID-19 pandemic, which altered educational and administrative processes globally. ^8^ By examining TE in this context, the study provides a comprehensive evaluation of program efficiency at a leading Peruvian institution and focus on postgraduate dental education in Peru, an underexplored area despite its relevance to educational policy and public health.

## Methods

### Study design

For this study, a cross-sectional design was proposed, with the population consisting of all document records submitted for obtaining a title of second professional specialty in each of the programs offered by the Postgraduate and Specialization Unit of the Dental School in the Universidad Peruana Cayetano Heredia (UPCH) in Lima, Peru, published in the institutional repository, a digital archive of academic documents, between 2017 and 2023. The decision was made to work with the entire population; therefore, no sample size was determined.

The study encompassed records of all students enrolled in second professional specialty programs at UPCH from 2017 to 2023, subject to specific selection criteria. Inclusion required complete documentation of program admission date, academic status, and graduation date for those who completed the program. Conversely, records with incomplete or inconsistent information in the above fields were excluded. Furthermore, the study omitted records of exchange students and those who transferred from other universities during the specified period. As all 258 records met the inclusion criteria, the entire population was analyzed, eliminating sampling bias.

### Variables

This research considered terminal efficiency (TE) as both a quantitative and qualitative variable. Additionally, some covariates were included, such as the year of formal document submission, the mode of document preparation, the second professional specialty in dentistry, and sex.

TE, as a quantitative variable or mean TE, was calculated from the difference in years between the formal submission of the document to obtain the title of second professional specialty to an expert jury, verified with the publication in the institutional repository of UPCH, and the student’s graduation year. As a qualitative variable or categorized TE, two categories were considered: “Up to 1 year” when TE was less than or equal to 1, and “2 years or more” when TE was greater than or equal to 2.

The mode of document preparation, as a qualitative variable, considered two categories: “Individual” when the document to obtain the title of second professional specialty in dentistry was prepared by a single student, and “Collective” when the document was prepared by two students or more.

The second professional specialty in dentistry, as a qualitative variable, considered twelve categories: “Dental Auditing”, “Oral and Maxillofacial Surgery”, “Endodontics”, “Special Patients Stomatology”, “Integral Oral Implantology”, “Restorative and Esthetic Dentistry”, “Pediatric Dentistry”, “Orthodontics and Maxillary Orthopedics”, “Periodontics and Implantology”, “Oral and Maxillofacial Radiology”, “Oral Rehabilitation”, and “Dental Public Health” which are part of the Peruvian list of recognized dental specialties in Article 26th of the Regulation of the Dentist’s Labor Law, Law No. 27878, amended in 2020 by Supreme Decree No. 023-2020-SA. ^9^

### Data collection

Data were sourced from the UPCH institutional repository, a public digital archive of documents submitted for second professional specialty titles (2017–2023). All 258 records meeting the inclusion criteria (complete admission, status, and graduation data) were extracted, with no exclusions, ensuring no selection bias.

Two researchers extracted data (sex, year of submission, mode of preparation, specialty) from repository metadata and full-text documents, using a structured protocol. Graduation years were obtained via a formal request to the repository administration. Data were recorded in Microsoft Excel 365 with unique identifiers for anonymity.

During cleaning, data completeness was verified by confirming that all records contained values for each variable (sex, year, mode, specialty, graduation year). No missing or incomplete data were identified, as all records met the inclusion criteria and repository entries were fully documented. To ensure this, the researchers cross-checked each record against metadata and full-text files, resolving any discrepancies through consensus or consultation with a third researcher. Standardized formats were applied (e.g., dates as YYYY, specialty names per Law No. 27878).

A random 10% sample (n=26) was cross-checked against original repository entries, and graduation years were validated with UPCH academic records. No missing data or inconsistencies were found, confirming the dataset's completeness. Data were anonymized per ethical approval (CONSTANCIA-CIEI-428-39-23).

### Data analysis

Sensitivity analyses were conducted to ensure the robustness of the findings. Outliers were identified using the interquartile range method as TE values below -3 years or above 5 years (Q1 − 1.5 × IQR or Q3 + 1.5 × IQR). ^10^ One record with a mean TE of 6 years was excluded, but its removal did not alter the direction or significance of the findings, confirming the robustness of the results. No missing or incomplete data were identified, as all 257 records met the inclusion criteria and were fully documented in the repository.

The descriptive analysis provided absolute and relative. For the bivariate analysis, associations were evaluated using the Chi-square statistical test; additionally, the Kolmogorov-Smirnov test was used to assess the normality of the data distribution (p < 0.001), and the non-parametric Mann-Whitney U and Kruskal-Wallis tests, with the corresponding post hoc test, were employed to determine differences between groups.

The chi-square test was used to determine if there is a significant association between two categorical variables. ^11^ The Mann-Whitney U test was employed to identify differences between the summary measures (medians) of dichotomous covariate categories (mode of document preparation and sex), as these categories divided the sample into two groups with independent measures. The Kruskal-Wallis test was used to detect differences between the summary measures (medians) of polytomous covariate categories (year of formal document submission and second professional specialty in dentistry), which divided the sample into more than two groups with independent measures. ^12^

For multivariate analysis, Poisson regression was employed to estimate crude (PR) and adjusted prevalence ratios (aPR). This method is primarily used for modeling count data, where the response variable represents counts of events, and it is useful for binary outcome analysis to estimate adjusted risk and prevalence ratios. ^13, 14^ In cross-sectional studies, it is often preferable to estimate prevalence ratios rather than odds ratios, especially when the outcome is not rare. ^14^ Two models were generated: a crude model (with year of formal document submission as the independent variable and categorized TE as the dependent variable) and an adjusted model incorporating covariates (mode of document preparation, second professional specialty in dentistry, and sex).

Additionally, a hierarchical multiple linear regression was developed; this statistical method analyzes data with a hierarchical structure, allowing for the inclusion of predictor variables at multiple levels of analysis while considering variance at each level. This method examines a continuous dependent variable to elucidate relationships between predictors and the dependent variable. ^15^ Given these characteristics, a variance inflation factor (VIF) test was performed to assess multicollinearity among the covariates. The results indicated values of less than 5 (VIF < 5), confirming that it was unnecessary to eliminate any variables. Consequently, the hierarchical multiple linear regression was deemed suitable for this study, facilitating the construction of models relating the independent variables to mean TE, as analyzed across the entire dataset.

Before this analysis, a logarithmic transformation was applied to the dependent variable due to its lack of normal distribution, which constrained the use of multiple linear regression as part of the proposed analytical statistics for this study. It is important to note that the application of logarithmic transformation is supported in various investigations, including those by Feng et al. in 2013, and Habibzadeh in 2024, which indicate that transforming a dataset with a non-normal distribution into one approximating normality is preferable, as statistical tests assuming normality typically yield more efficient inferences. ^16, 17^

The dataset with 257 records, as no missing or incomplete data were identified during extraction and cleaning, ensuring a complete dataset for all variables (sex, year, mode, specialty, graduation year), was imported into STATA v. 18.0 for analysis. Microsoft Excel 365 was used to organize and present the results in tables. The study considered a 95% confidence level and a p-value <0.05 to determine statistical significance.

## Results

The terminal efficiency (TE) of 257 Peruvian university students enrolled in second specialty programs at the dental school of Universidad Peruana Cayetano Heredia from 2017 to 2023 was analyzed. All records, sourced from the institutional repository, contained complete data with no exclusions due to missing or inconsistent information. Categorically, TE was achieved within one year by 72.37% of students (n = 186), while 27.63% (n = 71) required two years or more ( Table 1). The mean TE was calculated as 0.93 years (SD = 1.22), with a median of 1.00 year (IQR = 2.00) ( Table 2).

**Table 1.** Categorized terminal efficiency of Peruvian university students in the second professional specialty programs of a dental school over seven years.

Variables		n	%	Categorized terminal efficiency				
				Up to 1 year		2 years and older		p
				n	%	n	%	
Total		257	100.00	186	72.37	71	27.63	
Year of formal document submission								
	2017	25	9.73	21	84.00	4	16.00	0.067 *
	2018	33	12.84	30	90.91	3	9.09	
	2019	42	16.34	25	59.52	17	40.48	
	2020	54	21.01	39	72.22	15	27.78	
	2021	39	15.18	28	71.79	11	28.21	
	2022	29	11.28	19	65.52	10	34.48	
	2023	35	13.62	24	68.57	11	31.43	
Mode of document preparation								
	Individual	155	60.31	111	71.61	44	28.39	0.737 *
	Collective	102	39.69	75	73.53	27	26.47	
Second professional specialty in dentistry								
	Dental Auditing	2	0.78	2	100.00	0	0.00	0.562 **
	Oral and Maxillofacial Surgery	8	3.11	3	37.50	5	62.50	
	Endodontics	28	10.89	22	78.57	6	21.43	
	Special Patients Stomatology	7	2.72	5	71.43	2	28.57	
	Integral Oral Implantology	2	0.78	1	50.00	1	50.00	
	Restorative and Esthetic Dentistry	23	8.95	18	78.26	5	21.74	
	Pediatric Dentistry	31	12.06	24	77.42	7	22.58	
	Orthodontics and Maxillary Orthopedics	21	8.17	18	85.71	3	14.29	
	Periodontics and Implantology	17	6.61	6	35.29	11	64.71	
	Oral and Maxillofacial Radiology	53	20.62	48	90.57	5	9.43	
	Oral Rehabilitation	36	14.01	21	58.33	15	41.67	
	Dental Public Health	29	11.28	18	62.07	11	37.93	
Sex								
	Female	156	60.70	112	71.79	44	28.21	0.797 *
	Male	101	39.30	74	73.27	27	26.73	

n: Absolute frequency. %: Relative frequency. p: Statistical significance.

* Chi-square test.

** Chi-square test corrected by Yates.

**Table 2.** Mean terminal efficiency of Peruvian university students in the second professional specialty programs of a dental school over seven years.

Variables		n	%	Mean terminal efficiency				
				X	SD	M	IQR	p
Total		257	100.00	0.93	1.22	1.00	2.00	
Year of formal document submission								
	2017	25	9.73	0.48	1.08	0.00abcd	1.00	0.001 *
	2018	33	12.84	0.33	0.78	0.00efghi	0.00	
	2019	42	16.34	1.14	0.93	1.00aej	2.00	
	2020	54	21.01	0.74	1.01	0.00fj	2.00	
	2021	39	15.18	1.08	1.24	1.00bg	2.00	
	2022	29	11.28	1.34	1.47	1.00ch	2.00	
	2023	35	13.62	1.37	1.61	1.00di	3.00	
Mode of document preparation								
	Individual	155	60.31	0.91	1.32	0.00	2.00	0.141 **
	Collective	102	39.69	0.97	1.05	1.00	2.00	
Second professional specialty in dentistry								
	Dental Auditing	2	0.78	1.00	0.00	1.00a	0.00	<0.001 *
	Oral and Maxillofacial Surgery	8	3.11	2.00	1.60	2.00bcde	3.00	
	Endodontics	28	10.89	0.75	1.00	1.00bfgh	1.00	
	Special Patients Stomatology	7	2.72	1.43	1.51	1.00i	3.00	
	Integral Oral Implantology	2	0.78	1.50	0.71	1.50j	1.00	
	Restorative and Esthetic Dentistry	23	8.95	0.57	1.31	0.00cklmn	1.00	
	Pediatric Dentistry	31	12.06	1.03	1.20	1.00kop	1.00	
	Orthodontics and Maxillary Orthopedics	21	8.17	0.62	1.20	0.00dqr	1.00	
	Periodontics and Implantology	17	6.61	1.76	0.97	2.00floqs	1.00	
	Oral and Maxillofacial Radiology	53	20.62	0.30	0.97	0.00aegijpstu	0.00	
	Oral Rehabilitation	36	14.01	1.31	0.82	1.00hmrt	1.00	
	Dental Public Health	29	11.28	1.28	1.49	1.00nu	2.00	
Sex								
	Female	156	60.70	0.95	1.19	1.00	2.00	0.571 **
	Male	101	39.30	0.91	1.26	0.00	2.00	

n: Absolute frequency; %: Relative frequency; X: Mean; SD: Standard deviation; M: Median; IQR: Interquartile range; p: Statistical significance.

* Kruskal-Wallis test; post hoc Mann Whitney U test, equal letters show a statistically significant difference (p<0.05).

** Mann Whitney U test.

**Figure 1.**
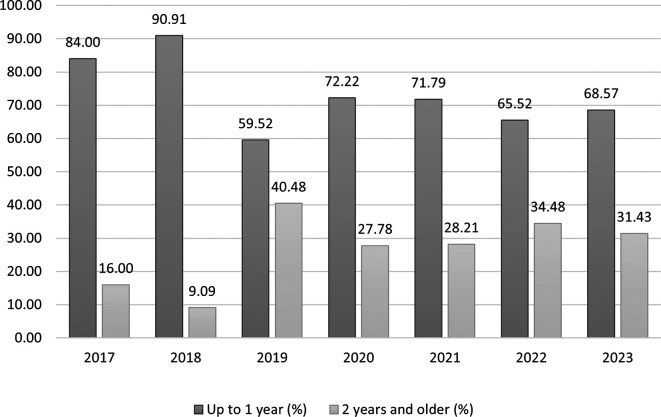
Categorized terminal efficiency of Peruvian university students in the second professional specialty programs of a dental school by year of formal document submission.

Associations between categorized TE and covariates were assessed using the Chi-square test. A significant association was identified with the year of document submission (χ ^2^ = 11.77, p = 0.067, Cramer's V = 0.21), though the p-value approached but did not reach the conventional threshold of 0.05. The proportion of students achieving TE within one year peaked in 2018 (90.91%, n = 30/33) and was lowest in 2019 (59.52%, n = 25/42) ( Figure 1 and Table 1). No significant associations were observed with the mode of document preparation (p = 0.737) or sex (p = 0.797) ( Table 1).

**Figure 2.**
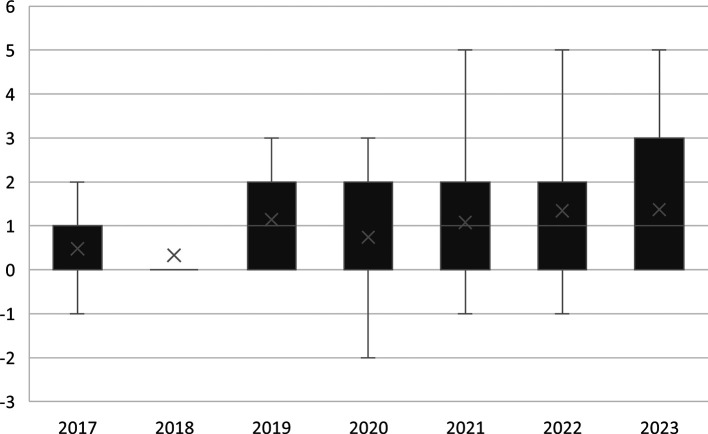
Boxplot of mean terminal efficiency of Peruvian university students in the second professional specialty programs of a dental school by year of formal document submission.

**Figure 3.**
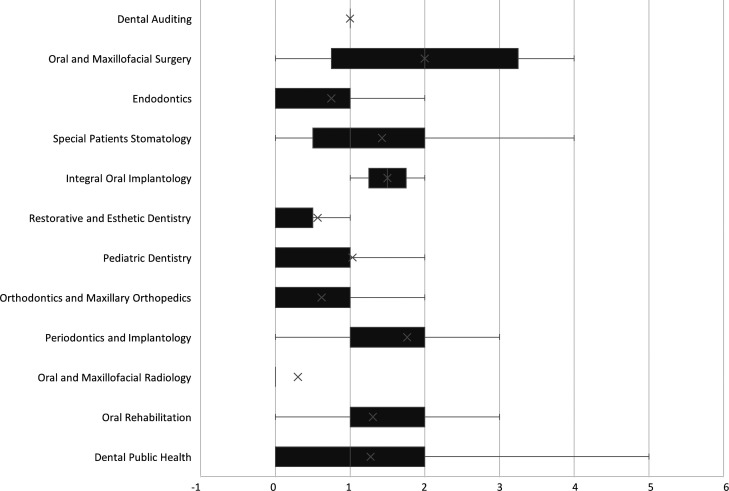
Boxplot of mean terminal efficiency of Peruvian university students over seven years by second professional specialty in dentistry.

Differences in mean TE across groups were evaluated using non-parametric tests due to the non-normal distribution of TE (Kolmogorov-Smirnov test, p < 0.001). The Kruskal-Wallis test revealed significant variation by year of submission (H = 24.08, p = 0.001, η ^2^ = 0.07), with post hoc Mann-Whitney U tests indicating that mean TE in 2018 (0.33 years) was significantly lower than in 2019 (1.14 years, p < 0.001), 2020 (0.74 years, p = 0.032), 2021 (1.08 years, p = 0.002), 2022 (1.34 years, p = 0.001), and 2023 (1.37 years, p < 0.002) ( Figure 2 and Table 2). Similarly, significant differences were detected across dental specialties (H = 55.87, p < 0.001, η ^2^ = 0.18). Post hoc tests showed that Oral and Maxillofacial Radiology (mean TE = 0.30 years) had a significantly lower TE compared to Dental Auditing (1.00 years, p = 0.029), Oral and Maxillofacial Surgery (2.00 years, p < 0.001), Endodontics (0.75 years, p = 0.002), Special Patients Stomatology (1.43 years, p = 0.004), Integral Oral Implantology (1.50 years, p = 0.016), Pediatric Dentistry (1.03 years, p < 0.001), Periodontics and Implantology (1.76 years, p < 0.001), Oral Rehabilitation (1.31 years, p < 0.001) and Dental Public Health (1.28 years, p < 0.001) ( Figure 3 and Table 2). No significant differences in mean TE were found by mode of document preparation (p = 0.141) or sex (p = 0.571) ( Table 2).

Poisson regression was employed to estimate prevalence ratios (PR) for categorized TE, adjusting for potential confounders (year of formal document submission, mode of document preparation, second professional specialty in dentistry, and sex). In the adjusted model, no significant predictors of achieving TE within one year were identified (p > 0.05 for all variables) ( Table 3). Hierarchical multiple linear regression was conducted on log-transformed mean TE to address its skewed distribution. The final model, adjusted for all covariates, explained 6% of the variance (R ^2^ = 0.06, p = 0.004), with the year of formal document submission as the only significant predictor (β = 0.144, 95% CI: 0.071–0.218, p < 0.001) ( Table 4).

**Table 3.** Multivariate analysis of the categorized terminal efficiency among Peruvian university students in the second professional specialty programs of a dental school over seven years.

Variables		Categorized terminal efficiency (Up to 1 year)					
		Crude model			Adjusted model		
		PR	95% CI	p	aPR	95% CI	p
Year of formal document submission							
	2017	Ref.			Ref.		
	2018	1.08	0.88-1.33	0.444	1.04	0.84-1.28	0.734
	2019	0.71	0.52-0.96	0.026	0.96	0.74-1.23	0.730
	2020	0.86	0.68-1.09	0.213	0.98	0.65-1.48	0.927
	2021	0.86	0.66-1.11	0.238	0.71	0.49-1.04	0.075
	2022	0.78	0.57-1.07	0.122	0.95	0.63-1.41	0.783
	2023	0.82	0.62-1.08	0.159	0.99	0.72-1.35	0.937
Mode of document preparation							
	Individual	Ref.					
	Collective	1.03	0.88-1.20	0.735	-	-	-
Second professional specialty in dentistry							
	Dental Auditing	Ref.					
	Oral and Maxillofacial Surgery	0.38	0.15-0.92	0.032	-	-	-
	Endodontics	0.79	0.65-0.95	0.015	-	-	-
	Special Patients Stomatology	0.71	0.45-1.14	0.159	-	-	-
	Integral Oral Implantology	0.50	0.13-2.00	0.327	-	-	-
	Restorative and Esthetic Dentistry	0.78	0.63-0.97	0.026	-	-	-
	Pediatric Dentistry	0.77	0.64-0.94	0.008	-	-	-
	Orthodontics and Maxillary Orthopedics	0.86	0.72-1.02	0.084	-	-	-
	Periodontics and Implantology	0.35	0.19-0.67	0.002	-	-	-
	Oral and Maxillofacial Radiology	0.91	0.83-0.99	0.025	-	-	-
	Oral Rehabilitation	0.58	0.44-0.77	<0.001	-	-	-
	Dental Public Health	0.62	0.47-0.83	0.001	-	-	-
Sex							
	Female	Ref.					
	Male	1.02	0.88-1.19	0.795	-	-	-

PR: Prevalence ratio; aPR: Adjusted prevalence ratio; 95% CI: 95% Confidence interval; p: Statistical significance.

a: Adjusted for mode of document preparation, second professional specialty in dentistry, and sex.

**Table 4.** Multivariate analysis of the mean terminal efficiency among Peruvian university students in the second professional specialty programs of a dental school over seven years.

Variables		R ^2^	Change of R ^2^	p-value Change of R ^2^	β _0_	β	β*	95% CI	p-value	p-value Model
Model 1		0.06	0.06	<0.001	0.404					<0.001
	Year of formal document submission					0.142	0.240	0.071-0.213	<0.001	
Model 2		0.06	0.00	0.866	0.377					0.001
	Year of formal document submission					0.140	0.237	0.068-0.213	<0.001	
	Mode of document preparation					0.023	0.011	-0.250-0.297	0.866	
Model 3		0.06	0.00	0.803	0.323					0.002
	Year of formal document submission					0.141	0.238	0.068-0.214	<0.001	
	Mode of document preparation					0.029	0.013	-0.248-0.305	0.839	
	Second professional specialty in dentistry					0.006	0.015	-0.038-0.049	0.803	
Model 4		0.06	0.00	0.442	0.484					0.004
	Year of formal document submission					0.144	0.244	0.071-0.218	<0.001	
	Mode of document preparation					0.011	0.005	-0.269-0.292	0.936	
	Second professional specialty in dentistry					0.005	0.015	-0.039-0.049	0.815	
	Sex					-0.106	-0.048	-0.378-0.166	0.442	

R ^2^: Determination coefficient; β0: Constant; β: Nonstandardized regression coefficient; β*: Standardized regression coefficient; 95% CI: 95% Confidence interval; p: Statistical significance.

## Discussion

The present study provides valuable insights into the terminal efficiency (TE) of Peruvian university students enrolled in second specialty programs in dentistry at a single institution over a seven-year period (2017–2023). The findings indicate an average TE of 0.93 years, with 72.37% of students completing their programs within ≤1 year of document submission and 27.63% requiring ≥2 years. These results suggest a generally efficient degree completion process within Peru’s postgraduate dental education framework, as governed by University Law 30220, which mandates a minimum of two academic semesters and a thesis or academic paper for second professional specialty titles. ^7^ However, significant variations in TE were observed across dental specialties (e.g., Oral Radiology at 0.30 years vs. Periodontics at 1.76 years) and years of document submission (lowest in 2018, highest in 2023), underscoring the influence of specialty-specific demands and temporal factors on program completion times. Multivariate analysis identified the year of document submission as a significant predictor of mean TE, albeit with limited explanatory power.

Comparative analysis with existing literature reveals both consistencies and divergences. Locally, Girano-Arévalo et al. (2021) reported a higher TE of 1.67 years for undergraduate dental students at the same institution (Universidad Peruana Cayetano Heredia, UPCH), with 60.51% completing in ≤1 year, ^5^ while Carrizales-Poma et al. (2024) found a TE of 1.52 years for UPCH master’s and doctoral dental programs. ^18^ The lower TE in our postgraduate cohort may reflect greater student commitment or streamlined research processes at the specialty level. Regionally, Costa Rican undergraduate dental students exhibited far lower efficiency, with only 6–8.5% completing in ≤1 year across multiple cohorts, ^8, 19^ suggesting that Peru’s postgraduate dental programs outperform some Latin American undergraduate programs. Globally, Mexican studies during the COVID-19 pandemic reported disrupted graduation rates, ^20^ while Chinese postgraduate medical students faced similar delays. ^21^ These comparisons highlight the relative efficiency of this study population, though contextual differences in program structure and external disruptions limit direct equivalence.

The research period (2017–2023) overlaps with the COVID-19 pandemic, which likely influenced TE trends, particularly from 2020 onward. The highest TE (1.76 years) in 2023 suggests prolonged completion times for documents submitted during or post-pandemic, potentially due to backlogs or persistent administrative delays. International parallels reinforce this hypothesis: Lange et al. (2023) noted greater graduation delays among United States postgraduate students compared to Swedish peers during the pandemic, ^22^ while Latin American studies identified barriers such as limited digital literacy and connectivity ^23^ and negative impacts on student well-being. ^24^ In Peru, the shift to remote learning and virtual thesis defenses ^25^ may have differentially affected specialties, with research-intensive fields like Periodontics potentially facing greater disruption than others like Oral Radiology. These findings suggest that pandemic-related challenges contributed to TE variability.

Practically, the specialty-specific TE disparities—Oral Radiology’s 0.30-year mean versus Periodontics’ 1.76 years—carry substantial implications for educational policy and administration. With an effect size indicating a 1.46-year difference, these variations likely stem from differences in research complexity, clinical requirements, or resource availability (e.g., imaging facilities vs. surgical training). Institutions could address lagging specialties through targeted interventions, such as structured mentorship programs or methodological workshops for Periodontics students, mirroring successful undergraduate strategies. ^5^ Notably, all submissions in this study were theses, despite UPCH regulations permitting academic papers. ^26^ So that, introducing hybrid thesis formats, as proposed elsewhere, ^27^ could reduce bottlenecks while aligning with Law 30220’s flexibility. ^7^ Such adaptations could enhance TE across specialties, balancing academic rigor with efficiency, and warrant pilot testing at the institutional level.

Despite its contributions, this study has limitations. Its single-institution focus at UPCH restricts generalizability to other Peruvian or Latin American contexts, where resource availability and student demographics may differ. The cross-sectional design, while robust for descriptive analysis, precludes causal inferences regarding covariates like specialty or submission year, and unexamined factors (e.g., sociodemographic profiles, institutional support) may also influence TE. Future multi-institutional and longitudinal studies tracking regional student cohorts could elucidate predictive factors and pandemic-specific effects. ^4, 8^ Multicenter investigations across Peru and Latin America ^20^ would further contextualize findings, while pre- and post-COVID-19 cohort comparisons could quantify long-term disruptions. ^22, 24^ These approaches would strengthen the evidence base for optimizing TE in dental education.

Actionable steps for stakeholders emerge from these insights. Institutions should enhance digital infrastructure to support remote learning and research continuity, mitigating future disruptions as seen during COVID-19. ^23, 28^ Policymakers could standardize TE metrics within regional accreditation frameworks, fostering consistency across Latin America. ^7, 8^ Specialty-specific support—e.g., tailored supervision for high-TE fields like Periodontics—should be prioritized, alongside incentives for hybrid thesis formats under existing legal provisions. ^7, 26^ Collectively, these measures could optimize TE, aligning educational efficiency with Peru’s scientific and professional development goals. ^29^

In conclusion, this study provides a detailed evaluation of terminal efficiency (TE) among Peruvian university students in second specialty dental programs at a single institution from 2017 to 2023. Most students achieved TE within one year, with a mean completion time under one year, though significant variations emerged across submission years and dental specialties. Temporal analysis revealed that the year of document submission significantly influenced mean TE, with longer completion times in later years, particularly post-2020, likely reflecting the impact of the COVID-19 pandemic. Specialty-specific differences were also pronounced, with some programs demonstrating markedly shorter completion times than others, suggesting variations in research complexity or resource demands. In contrast, neither the mode of document preparation nor sex significantly affected TE, indicating that these factors may not be primary drivers of efficiency in this context.

These findings highlight the resilience of the studied postgraduate framework amidst external disruptions, while identifying areas for improvement, such as specialty-specific support to address prolonged completion times and can guide targeted interventions to optimize postgraduate dental education, supporting Peru’s goals for advancing health sciences training.

### Ethical considerations

The study protocol was approved by the UPCH Institutional Research Ethics Committee (ethics file CONSTANCIA-CIEI-428-39-23, approved on October 2, 2023). All extraction and cleaning processes adhered to ethical guidelines, using anonymized data from public repository sources.

### Consent to participate

Consent to participate was not required, due to the study used secondary data, which was anonymized, ensuring that such modification does not distort the scientific meaning of the information. An accredited committee, the Universidad Peruana Cayetano Heredia Institutional Research Ethics Committee approved the study protocol in the “Exempt” category, which exempts the study protocol from expedited review and the need for consent to participate because the study uses information that is public in the university’s institutional repository.

## Author contributions

Karen Llajaida Cabanillas-Yllesca

Roles: Conceptualization, Methodology

Karla Lucero Avalos-Baltodano

Roles: Investigation, Resources

Roberto A. León-Manco

Roles: Conceptualization, Methodology, Writing – Review & Editing

María Claudia Garcés-Elías

Roles: Conceptualization, Methodology, Writing – Review & Editing

Cesar D. Rojas-Senador

Roles: Data Curation, Formal Analysis, Investigation, Writing – Review & Editing

## Data availability

Zenodo: Terminal efficiency of Peruvian university students in the second specialty programs of a dental school over seven years - Dataset. https://doi.org/10.5281/zenodo.13901816. ^30^

The project contains the following underlying data:

• Dataset-Terminal efficiency.xls. (Anonymised and codified data, with legend).

Data are available under the terms of the Creative Commons Attribution 4.0 International license (CC-BY 4.0).

### Reporting guidelines

Zenodo: STROBE checklist for “Terminal efficiency of Peruvian university students in the second specialty programs of a dental school over seven years”. https://doi.org/10.5281/zenodo.13939895. ^31^

Data are available under the terms of the Creative Commons Attribution 4.0 International license (CC-BY 4.0).
