# Supplementary material for: Terminal efficiency of Peruvian university students in the second specialty programs of a dental school over seven years
Source: F1000Res. 2025 May 9;13:1307. Originally published 2024 Oct 31. [Version 2] doi: 10.12688/f1000research.157705.2 (PMC12123260; doi:10.12688/f1000research.157705.2)
Supplement: Peer review history

# Reviewer report: Reviewer response for version 2

**DOI:** 10.5256/f1000research.181537.r385055

**Published:** 2025-05-29

**Authors:** Dharmashree Satyarup, Manish Kumar

**Affiliations:** 1 Department of Public Health Dentistry, Institute of Dental Sciences, Siksha ‘O’ Anusandhan (Deemed to be University), Bhubaneswar, Odisha, India; 2 GITAM, Visakhapatnam, Andhra Pradesh, India

The manuscript is clearly and accurately presented. The authors have structured the work in a coherent and logical manner, allowing the reader to follow the progression of the study with ease. 

 The study design is cross-sectional but backed by relevant regression analysis to demonstrate the associations.

 Sufficient methodological details are provided to allow for independent replication of the study.

 The statistical analyses employed in the study are appropriate for the type and scale of data collected. The authors provide clear explanations of the statistical methods used, including the rationale for their selection.

 The conclusions drawn are well-supported by the results. The authors provide a balanced discussion, considering the implications of their findings within the context of the field, and recommendations are stated based on the findings of the study conducted for relevant stakeholders.

Is the work clearly and accurately presented and does it cite the current literature?

Yes

If applicable, is the statistical analysis and its interpretation appropriate?

Yes

Are all the source data underlying the results available to ensure full reproducibility?

Yes

Is the study design appropriate and is the work technically sound?

Yes

Are the conclusions drawn adequately supported by the results?

Yes

Are sufficient details of methods and analysis provided to allow replication by others?

Yes

Reviewer Expertise:

Oral health promotion, oral health education, prevention

We confirm that we have read this submission and believe that we have an appropriate level of expertise to confirm that it is of an acceptable scientific standard.

# Reviewer report: Reviewer response for version 2

**DOI:** 10.5256/f1000research.181537.r383879

**Published:** 2025-05-16

**Authors:** Avita Rath

**Affiliations:** 1 SEGi University, Kota Damansara, Malaysia

I appreciate the authors' comprehensive effort to revise the manuscript in accordance with the earlier peer review. The improvements in methodological transparency, statistical rationale, and contextual analysis are commendable. The enhanced clarity in the **Introduction** and **Discussion**, inclusion of **multivariate analyses**, and refined **figures and tables** significantly strengthen the paper’s scientific rigor and educational relevance.

 Specifically:

- The **Methods** section is now much more replicable, with clear articulation of the data extraction, cleaning, verification, and statistical procedures.
- The **Discussion** more thoroughly addresses the implications of COVID-19, specialty-specific disparities, and cross-regional relevance.
- The **Conclusions** are appropriately tempered to reflect the exploratory nature of the study and now align better with the presented data.

The revisions align with the earlier recommendations, and I find the current version suitable for indexing.

Is the work clearly and accurately presented and does it cite the current literature?

Partly

If applicable, is the statistical analysis and its interpretation appropriate?

Partly

Are all the source data underlying the results available to ensure full reproducibility?

Yes

Is the study design appropriate and is the work technically sound?

Partly

Are the conclusions drawn adequately supported by the results?

Partly

Are sufficient details of methods and analysis provided to allow replication by others?

Partly

Reviewer Expertise:

NA

I confirm that I have read this submission and believe that I have an appropriate level of expertise to confirm that it is of an acceptable scientific standard.

# Reviewer report: Reviewer response for version 1

**DOI:** 10.5256/f1000research.173196.r365454

**Published:** 2025-02-12

**Authors:** Avita Rath

**Affiliations:** 1 SEGi University, Kota Damansara, Malaysia

**Summary of the Article:**

 This study evaluates the terminal efficiency (TE) of Peruvian university students enrolled in second specialty programs at a dental school over a seven-year period (2017-2023). Using a cross-sectional design, the authors analyzed all available records from the institutional repository of Universidad Peruana Cayetano Heredia. Terminal efficiency was assessed both quantitatively (mean TE in years) and qualitatively (categorized TE as “Up to 1 year” or “2 years or more”). The study utilized descriptive statistics and bivariate analyses (Chi-square, Mann-Whitney U, and Kruskal-Wallis tests) to explore associations between TE and variables such as year of document submission, mode of document preparation, dental specialty, and sex.

**Overall Assessment:**

 The study addresses a relevant topic in dental education, particularly the efficiency of postgraduate programs. While the article is generally well-structured and the data are publicly available, several aspects of the methodology, statistical analysis, and interpretation require clarification and refinement to ensure the study is scientifically sound.

**1. Clarity and Accuracy of Presentation (Partly)**

- The manuscript is generally clear but could benefit from simplifying some complex sentences in the **Introduction** and **Discussion** sections to enhance readability.
- The **Results** section is dense with data, and incorporating more **visual aids** (e.g., graphs, detailed tables) would improve clarity and help readers better interpret the findings.
- While relevant literature is cited, the manuscript would benefit from including more **recent international studies** on terminal efficiency in postgraduate dental education to broaden its context.

**Recommendations:**

- Simplify overly complex sentences and enhance the flow of the Introduction and Discussion.
- Add more visual elements to the Results section to present key findings more effectively.
- Incorporate recent international studies to strengthen the literature review.

**2. Study Design and Technical Soundness (Partly)**

- The **cross-sectional design** is appropriate for the study’s aims, but it inherently limits causal inferences about factors influencing terminal efficiency.
- The study includes all available records, reducing selection bias. However, the analysis would be strengthened by incorporating **multivariate analysis** to control for potential confounders such as specialty, sex, and year of submission.

**Recommendations:**

- Consider performing a **multivariate regression analysis** to control for confounding variables.
- Clearly articulate the limitations of the cross-sectional design in the Discussion.

**3. Methods and Replicability (Partly)**

- While the methods are described in detail, some aspects need further clarification to ensure full replicability:
  - The **data collection process** should be described in more detail, particularly how data were **extracted, cleaned, and verified**.
  - The manuscript should specify how **missing data** were handled.
  - The **sensitivity analyses** are mentioned but lack detail on the specific procedures used.

**Recommendations:**

- Provide detailed descriptions of the data extraction, cleaning, and verification processes.
- Clarify how missing or incomplete data were addressed.
- Expand on the sensitivity analyses, including criteria for identifying outliers and how they were handled.

**4. Statistical Analysis and Interpretation (Partly)**

- The use of **Chi-square**, **Mann-Whitney U**, and **Kruskal-Wallis tests** is appropriate for the data; however, the rationale for selecting these tests over others should be explained.
- The manuscript focuses on statistical significance but does not discuss **effect sizes** or the **practical significance** of the findings.
- **Post hoc analyses** following the Kruskal-Wallis test are not sufficiently detailed.

**Recommendations:**

- Provide a clear rationale for the selection of specific statistical tests.
- Include **effect sizes** and discuss the **practical implications** of the findings.
- Expand on the **post hoc analyses**, detailing how they were conducted and interpreted.

**5. Data Availability and Reproducibility (Yes)**

- The manuscript meets the F1000Research standard for data availability. The dataset and STROBE checklist are publicly accessible via **Zenodo**, ensuring reproducibility.

**No action required.**

**6. Conclusions and Support from Results (Partly)**

- The conclusions are generally supported by the results, particularly regarding the mean terminal efficiency and its variation across specialties and submission years.
- However, the conclusions overgeneralize the findings without fully considering the study’s limitations (e.g., single-institution focus, lack of multivariate analysis).
- The impact of external factors like **COVID-19** is mentioned but not deeply analyzed in relation to the data.

**Recommendations:**

- Temper the conclusions to reflect the **exploratory nature** of the study and its **limitations**.
- Discuss the **practical implications** of the findings for educational policy and administration.
- Analyze the potential impact of **external factors** (e.g., COVID-19) more thoroughly.

**Final Recommendation:**

**Approved with Reservations**

 The study has academic merit and provides valuable insights into terminal efficiency in postgraduate dental education. However, addressing the methodological, statistical, and interpretative issues outlined above is necessary to ensure the manuscript meets the highest standards of scientific rigor.

 Thank you for your contributions to the field.

Is the work clearly and accurately presented and does it cite the current literature?

Partly

If applicable, is the statistical analysis and its interpretation appropriate?

Partly

Are all the source data underlying the results available to ensure full reproducibility?

Yes

Is the study design appropriate and is the work technically sound?

Partly

Are the conclusions drawn adequately supported by the results?

Partly

Are sufficient details of methods and analysis provided to allow replication by others?

Partly

Reviewer Expertise:

Health professional education, DEI, Scholarship of traching and learning, TEL

I confirm that I have read this submission and believe that I have an appropriate level of expertise to confirm that it is of an acceptable scientific standard, however I have significant reservations, as outlined above.
